# Short-Term Effect of Moderate Level Air Pollution on Outpatient Visits for Multiple Clinic Departments: A Time-Series Analysis in Xi’an China

**DOI:** 10.3390/toxics11020166

**Published:** 2023-02-09

**Authors:** Qingnan Wang, Zhuo Chen, Wei Huang, Bo Kou, Jingwei Li

**Affiliations:** 1 Department of Information Management, School of Management, Xi’an Jiaotong University, Xi’an 710049, China; 2 College of Public Health, University of Georgia, Athens, GA 30602, USA; 3 School of Economics, University of Nottingham Ningbo China, Ningbo 315000, China; 4 College of Business, Southern University of Science and Technology, Shenzhen 518055, China; 5 Department of Otolaryngology, the First Affiliated Hospital of Xi’an Jiaotong University, Xi’an 710016, China

**Keywords:** outpatient visits, moderate pollution, exposure–response effect, distributed nonlinear model, ophthalmology disease, pediatric department disease

## Abstract

There is limited evidence concerning the association between air pollution and different outpatient visits in moderately polluted areas. This paper investigates the effects of moderate-level air pollution on outpatient visits associated with six categories of clinic department. We analyzed a total of 1,340,791 outpatient visits for the pediatric, respiratory, ear-nose-throat (ENT), cardiovascular, ophthalmology, and orthopedics departments from January 2016 to December 2018. A distributed lag nonlinear model was used to analyze the associations and was fitted and stratified by age and season (central heating season and nonheating season). We found ${\mathrm{SO}}_{2}$ had the largest effect on pediatrics visits (RR = 1.105 (95%CI: 1.090, 1.121)). Meanwhile, ${\mathrm{PM}}_{2.5}$ and ${\mathrm{SO}}_{2}$ had greater effects on ENT visits for people under 50 years old. The results showed a strong association between $O_{3}$ and cardiovascular outpatient visits in the nonheating season (RR = 1.273, 95% CI: 1.189,1.358). The results showed every 10 $\mu g/m^{3}$ increase in ${\mathrm{SO}}_{2}$ was associated with a lower number of respiratory outpatient visits. Significant different associations were observed in ${\mathrm{PM}}_{2.5}$, ${\mathrm{NO}}_{2}$, *CO*, and $O_{3}$ on ophthalmology visits between the heating and nonheating seasons. Although no significant association has been found in existing studies, our findings showed ${\mathrm{PM}}_{2.5}$ and ${\mathrm{NO}}_{2}$ were significantly related to orthopedic outpatient visits for people under 60 (RR = 1.063 (95%CI: 1.032, 1.095), RR = 1.055 (95%CI: 1.011, 1.101)). This study also found that the effect-level concentrations of air pollutants for some clinic departments were lower than the national standards, which means that people should also pay more attention when the air quality is normal.

## 1. Introduction

Air pollution and climate change have become global health concerns. Particulate matter contributes to more hospital admissions [1,2], higher disease incidence [3], excess mortality [4,5], and increased stress in hospital management systems such as emergency ambulance dispatches [6,7]. Therefore, understanding the associations between air pollution and the incidence of various diseases can help vulnerable groups to take preventive measures in their daily lives.

The literature regarding the effect of air pollutants on health is mainly focused on respiratory and circulatory systems, with a few studying the effect on ophthalmology and internal medicine. Yao et al. discovered that 12,000 inpatients were attributed to ${\mathrm{PM}}_{2.5}$ per year, among which cardiovascular diseases accounted for 15.9–20.0% and respiratory diseases for 7.9–9.2%; outpatient visits related to ${\mathrm{PM}}_{2.5}$ (internal medicine and pediatrics) accounted for 3.9–5.4% of the total population in Shanghai [8]. Wang et al. analyzed glaucoma outpatient visits in Shenyang from 2017 to 2019 and observed that every 10 units of increase in ${\mathrm{PM}}_{10}$, ${\mathrm{SO}}_{2}$, and ${\mathrm{NO}}_{2}$ led to a 0.7%, 2.4%, and 4.1% increase in glaucoma outpatient visits, respectively [9]. Atkinson et al. demonstrated that particle concentrations in eight European cities were positively associated with increased numbers of admissions for respiratory diseases, based on the APHEA 2 project [10]. Cole et al. analyzed 355 cities in COVID during COVID-19 and found that an area with 1 $\mu g/m^{3}$ more ${\mathrm{PM}}_{2.5}$ contributed to 9.4 COVID-19 cases and 3 more hospital admissions [11]. Chau et al. observed that air pollution increased the susceptibility risk of 18 diseases by more than 40% of the daily diagnosis, including spondylosis, cerebrovascular ailments, pneumonia, accidents, chronic obstructive pulmonary disease, influenza, osteoarthritis, asthma, peptic ulcer disease, cancer, heart disease, hypertension, diabetes, kidney disease, and rheumatism [12]. Moreover, Seposo et al. discovered that $O_{3}$, ${\mathrm{NO}}_{2}$, and ${\mathrm{PM}}_{2.5}$ were positively correlated with visits of outpatients with cardiopulmonary disease during both a short and a longer lag period [13].

Air pollution’s adverse effect on health is a regional problem. Samoli et al. found that the differences in the pollutant combinations, population characteristics, and climate in different cities caused differences in the relationships between pollution and health effects [14]. Existing studies were primarily conducted in heavily polluted megacities such as Beijing [15], Shanghai [16], and New York [17]. However, few studies exist on the pathogenic mechanism of pollutants in areas or cities with moderate pollution levels, which are more common in developing countries like China. Because a growing body of evidence exists on the associations between low-level air pollution exposure and increased mortality [18,19], it is essential to fill the research gap in this field.

As observed, epidemiological studies commonly use mortality and morbidity to analyze the effects of air pollutants on health [20]. However, most hospitals in developing countries like China are appointment-free, and the patients are attended to on a first-come, first-served basis [21]. Therefore, outpatient visits are more suitably used to track actual morbidity in China more reliably than other measures.

This study collected outpatient visit data from the following departments: pediatrics, ENT (ear-nose-throat), cardiovascular, respiratory, ophthalmology, and orthopedics. Orthopedics was the control group because few studies have shown air pollutants are related to orthopedic diseases. Our findings are proposed to help hospital managers optimize clinic resource allocation according to air quality and to provide additional evidence for the government to determine air-quality standards.

## 2. Materials and Methods

### 2.1. Study Area and Population

**Figure 1:**
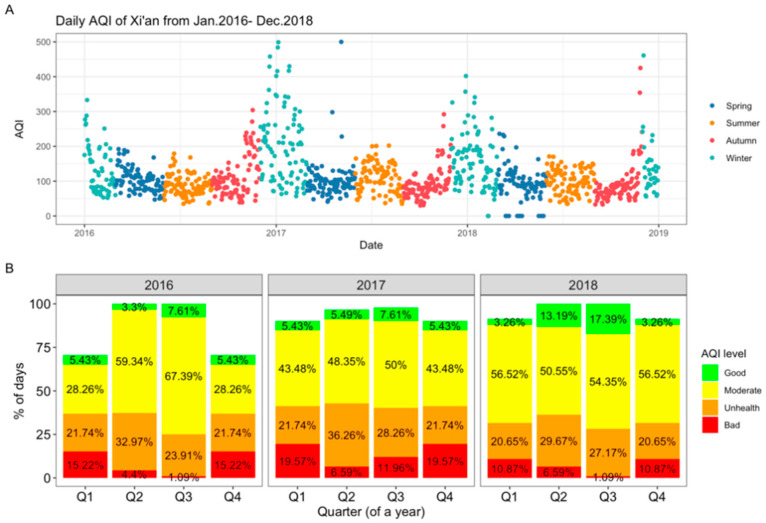
Time trend of air quality index (AQI) and quarterly AQI in Xi’an from Jan 2016 to Dec 2018. AQI level: Good (AQI: 0~50), Mild (AQI: 51~100), Moderate (AQI: 101~150), Heavy (AQI: >151). (**A**) Daily AQI of Xi’an from Jan 2016–Dec 2018; (**B**) Quarterly AQI level of Xi’an.

Xi’an, the largest city in northwestern China, with a permanent residential population of more than 12.95 million in 2020, is located at 34.2658° north latitude and 108.9541° east latitude. Xi’an has a temperate monsoon climate with four distinct seasons, and the annual average temperature is 13.6 ℃. It is classified as part of China’s central-heating area (north to the Qinling Mountains and the Huaihe River, with a centrally controlled public heating system). The heating season lasts from 15 November to 15 March. ${\mathrm{PM}}_{2.5}$ and ${\mathrm{PM}}_{10}$ are primary pollutants in Xi’an, while ${\mathrm{NO}}_{2}$ and $O_{3}$ have risen sharply in recent years. The daily AQI of Xi’an exhibits a seasonal shift; it is high in winter and spring and low in summer and autumn (Figure 1A). Xi’an’s air quality has improved significantly since “Shaanxi Provincial Atmospheric Pollution Prevention Regulations” was published in 2013. Figure 1b shows that within every quarter, Xi’an is classified as mildly polluted 50% to 60% of the time according to China and U.S. AQI standards [22,23].

### 2.2. Hospital Outpatient Visits Data

We collected outpatient visit data from a large hospital in Xi’an between 1 January 2016 and 31 December 2018. The target hospital is one of the largest hospitals in northwestern China, with 3.26 million outpatients and emergency visits in 2019. Patients with fixed addresses outside Xi’an were excluded. The hospital’s ethics committee approved the protocol of this study and the access to the hospital outpatient data (approval number: XJTU1AF2021LSK-2021-115).

### 2.3. Air Pollution and Meteorological Data

**Figure 2:**
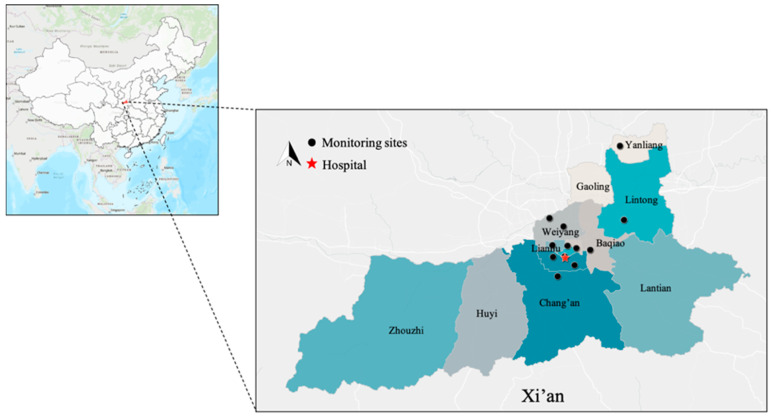
Locations of monitoring stations in Xi’an.

Air pollution data were collected at 13 environmental monitoring stations in Xi’an (Figure 2), established by the Xi’an Environmental Protection Administration. We took the average of the air pollution concentrations from the 13 monitoring stations as the final data. Air pollutants included ${\mathrm{PM}}_{2.5}$ ($\mu g/m^{3}$), ${\mathrm{PM}}_{10}$ ($\mu g/m^{3}$), ${\mathrm{SO}}_{2}$ ($\mu g/m^{3}$), ${\mathrm{NO}}_{2}$ ($\mu g/m^{3}$), CO ($\mathrm{mg}/m^{3}$), and $O_{3}$ ($\mu g/m^{3}$). Daily 24 h concentrations were calculated by averaging air pollutant concentrations over 24 h per day for all five pollutants except $O_{3}$, which was calculated as 8 h maximum values (from 10 a.m. to 6 p.m.). Missing data were imputed by linear interpolation. Daily meteorological data, including mean temperature (℃), minimum temperature (℃), maximum temperature (℃), and relative humidity (%), were collected from the Xi’an Meteorological Administration.

### 2.4. Analysis Method

A quasi-Poisson generalized additive model (GAM) combined with a distributed lag nonlinear model (DLNM) was built to illustrate the association between air pollution and outpatient visits. DLNM can analyze the nonlinear exposure–response relationship and capture the cumulative health risks for different air pollution exposure lag days than single-lag days or moving averages [24]. Previous studies have shown that ${\mathrm{PM}}_{10}$ has a 3–5 day lag effect on respiratory disease admission, ${\mathrm{SO}}_{2}$ has a 1–3 day lag effect on CVD mortality, and ${\mathrm{NO}}_{2}$ has a 1–4 day lag effect on respiratory disease admission. Therefore, we chose a maximum lag of 5 days in the DLNM for all air pollutants as a priori in the primary analyses [25].

A natural cubic spline cross-basis function was built to account for the potentially lagged and nonlinear effects of the daily mean temperatures, and the maximum lag days was 14 days (2 weeks) [26]. A natural cubic spline function for calendar date was used to control for seasonality or long-term trends. The degrees of freedom for time trend were determined by minimizing the sum of the absolute value of the partial autocorrelation function (PACF) of the model’s residuals up to 20 lags. We chose df as 3, 4, 7, 3, 2, 4 for the cardiovascular, ENT, ophthalmology, orthopedics, pediatrics, and respiratory departments, respectively. Dummy variables for day of the week (DOW) and public holidays were used to control for short-term effects. A natural cubic spline function with 3 df was used to control for the relative humidity (RH). The basic model was as follows:

(1) $$\log\left[E\left(Y_{t}\right)\right]=\alpha +\beta DLNM\left(X_{t},\;df=5\right)+DLNM\left(temperature,\;df=5\right)+ns(Calender\;Date,\;df=df*3)+\;ns\left(RH,\;df=3\right)+factor\left(DOW\right)+factor\left(Holiday\right)+\varepsilon ,$$

where $E\left(Y_{t}\right)$ indicates the expected number of outpatient visits at day t; α is the intercept; β represents the log-relative rate of outpatient visits associated with a unit increase in each pollutant concentration; $DLNM\left(X_{t}\right)$ is the quasi-Poisson DLNM for each air pollutant; DLNM(temperature) is the DLNM function for daily temperature; ns is the natural cubic spline function; *RH* is the relative humidity; *DOW* is a dummy variable representing the day of the week (Monday to Sunday); Holiday is a dummy variable representing public holidays to control for short-term fluctuations; and ε represents the residual error.

The relative risks (RRs) associated with every 10-unit increase in pollutants were calculated. Single-pollutant models were built to estimate each pollutant’s exposure–response relationship. Two-pollutant models were built by adding one pollutant at a time to test the robustness of each air pollutant’s effect on daily outpatient visits. Exposure–response curves were also fitted for the associations between air pollutants and outpatient visits.

Sensitivity analyses were conducted to test the robustness of the findings. DLNM controlled for longer effects of temperature, including maximum lags of 7, 14, and 21 days. Alternative dfs (4, 8, and 12 dfs per year) were adjusted for long-term trends.

All statistical tests were performed using R software (Version 3.6.4) with the “dlnm” package for the DLNM model and the “mgcv” package for the GAM model [27,28]. Statistical tests were two-sided, with the significance level set at *p*-value < 0.05.

## 3. Results

### 3.1. Descriptive Analysis and Correlation Analysis

**Figure 3:**
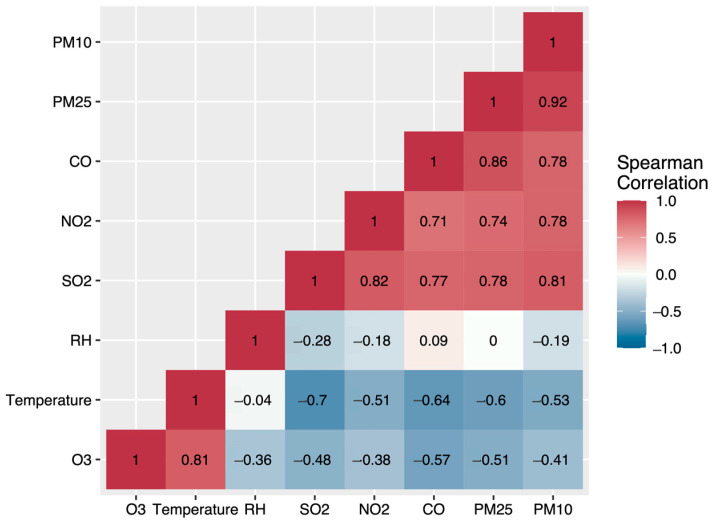
Thermogram of Spearman’s correlation coefficient matrix of weather conditions and air pollutants in Xi’an, 2016–2018.

Figure 3 shows strong correlations between pollutants, with Spearman’s correlation coefficients running from 0.71 to 0.93 (Table S1 showed the detailed values). All pollutants except for $O_{3}$ were negatively correlated with average temperature and RH, because $O_{3}$ was more sensitive to elevated temperatures.

Air pollutants showed distinct seasonal trends, with concentrations of ${\mathrm{PM}}_{2.5}$, ${\mathrm{PM}}_{10}$, ${\mathrm{SO}}_{2}$, ${\mathrm{NO}}_{2}$, and CO higher from November to May and lower from June to October, while $O_{3}$ showed the opposite trend because of its formation mechanism. During the study period, the concentrations of air pollutants exceeded China’s new National Meteorological Air Quality Standard Level II (NAAQS) (GB3095-2012) [11,23] for ${\mathrm{PM}}_{2.5}$ for 298 days (27.2%), ${\mathrm{PM}}_{10}$ for 282 days (25.7%), ${\mathrm{SO}}_{2}$ for 0 days, ${\mathrm{NO}}_{2}$ for 133 days (12.1%), CO for 9 days (0.8%), and $O_{3}$ for 156 days (14.23%). For meteorological factors, the average daily humidity and temperature levels during the study period were 66.12 ± 17.03 (%) and 15.59 ± 10.18 (℃), respectively.

There were a total of 1,577,807 outpatient visits during the study period. The daily average number of outpatient visits was 1512, of which 21.29% were pediatric, 15.93% were ENT, 17.79% were cardiovascular, 16.27% were respiratory, 12.90% were ophthalmologic, and 15.80% were orthopedic outpatients (Table 1). There were apparent seasonal trends for all departments except orthopedics.

### 3.2. Exposure-Effect Analysis

Table 2 shows the relative risks (RRs) of air pollutants for daily outpatient visits in each department, stratified by season and age. Overall, a 10-unit increase in the concentrations of ${\mathrm{PM}}_{2.5}$, ${\mathrm{PM}}_{10}$, ${\mathrm{SO}}_{2}$, ${\mathrm{NO}}_{2}$, CO, and $O_{3}$ was associated with RRs of all visits of 1.088, 1.035, 1.381, 1.137, 1.114, and 1.152, respectively.

Among all departments, the orthopedics department was the least affected by air pollution. No positive association was observed for changes in $O_{3}$. There were significantly stronger associations in the heating season for ${\mathrm{PM}}_{2.5}$, ${\mathrm{SO}}_{2}$, and CO. For different population groups, significant associations were observed for patients over 6 years old with ${\mathrm{PM}}_{2.5}$ and ${\mathrm{NO}}_{2}$ and for patients under 6 years old with ${\mathrm{PM}}_{10}$ and ${\mathrm{SO}}_{2}$. ${\mathrm{NO}}_{2}$ had significant associations in both age groups, but the effects were higher on the patients over 6 years old than those under 6 years old.

For ENT patients, a 10 $\mu g/m^{3}$ increase in the concentrations of ${\mathrm{PM}}_{2.5}$, ${\mathrm{PM}}_{10}$, ${\mathrm{SO}}_{2}$, ${\mathrm{NO}}_{2}$, and $O_{3}$ increased the risk of pediatric patient visits. For different seasons, ${\mathrm{PM}}_{10}$, ${\mathrm{NO}}_{2},$ and $O_{3}$ had significantly stronger associations in the heating season than in the nonheating season. For different population groups, significant associations were observed for the patients under 50 years old with ${\mathrm{PM}}_{2.5}$ and ${\mathrm{SO}}_{2}$ and for the patients over 50 years old with ${\mathrm{PM}}_{10}$.

For cardiovascular patients, an increase of 10 units in the concentration of CO was associated with a higher risk than other outpatients, followed by ${\mathrm{PM}}_{2.5}$. Additionally, ${\mathrm{PM}}_{2.5}$ was significantly associated with cardiovascular outpatient visits in the nonheating season. There was a significant difference in the adverse effects of ${\mathrm{PM}}_{10}\;$ and $O_{3}$ between the heating season and the nonheating season. After stratifying by age, the effects of ${\mathrm{NO}}_{2}$, CO, and $O_{3}$ on different age groups were significantly different.

For the respiratory department, an increase of 10 units in the concentration of ${\mathrm{PM}}_{2.5}$ had a higher risk for outpatient visits, followed by ${\mathrm{PM}}_{10}$ and $O_{3}$, which had similar effects. After stratifying by seasons, the effects of ${\mathrm{PM}}_{2.5}$, ${\mathrm{NO}}_{2}$, and CO on outpatient visits were significantly different between the heating and nonheating seasons. The results showed that ${\mathrm{PM}}_{2.5}$ had stronger associations in the nonheating season, whereas ${\mathrm{NO}}_{2}$ and CO had stronger associations in the heating season. For different age groups, ${\mathrm{NO}}_{2}$ was significantly associated with patients of all ages. ${\mathrm{SO}}_{2}$ and CO were significantly associated with the patients under 60 years old, and $O_{3}$ was significantly associated with the patients over 60 years old.

For the ophthalmology department, an increase of 10 units in the concentration of ${\mathrm{PM}}_{10}$ had a higher risk of outpatient visits than CO and ${\mathrm{PM}}_{2.5}$, whereas $O_{3}$ had a positive effect on ophthalmology outpatient visits. The results showed that ${\mathrm{PM}}_{2.5}$, ${\mathrm{NO}}_{2}$, CO, and $O_{3}$ had significantly different associations with ophthalmology visits between the heating and nonheating seasons. Significant associations were observed between the patients under 60 years old and ${\mathrm{SO}}_{2}$, CO, and $O_{3}$, and between the patients over 60 years old and ${\mathrm{PM}}_{2.5}$, ${\mathrm{NO}}_{2}$, and CO.

For orthopedics, an increase of 10 $\mu g/m^{3}$ in the concentrations of ${\mathrm{PM}}_{2.5}$ and ${\mathrm{NO}}_{2}$ was associated with an increased risk of outpatient visits. For ${\mathrm{NO}}_{2}$, the estimated RRs were significant for both seasons and slightly but not significantly stronger in the nonheating season. Significant associations with ${\mathrm{PM}}_{2.5}$ and ${\mathrm{NO}}_{2}$ were observed for patients under 60 years old and with $O_{3}$ and CO for patients over 60 years old.

**Figure 4:**
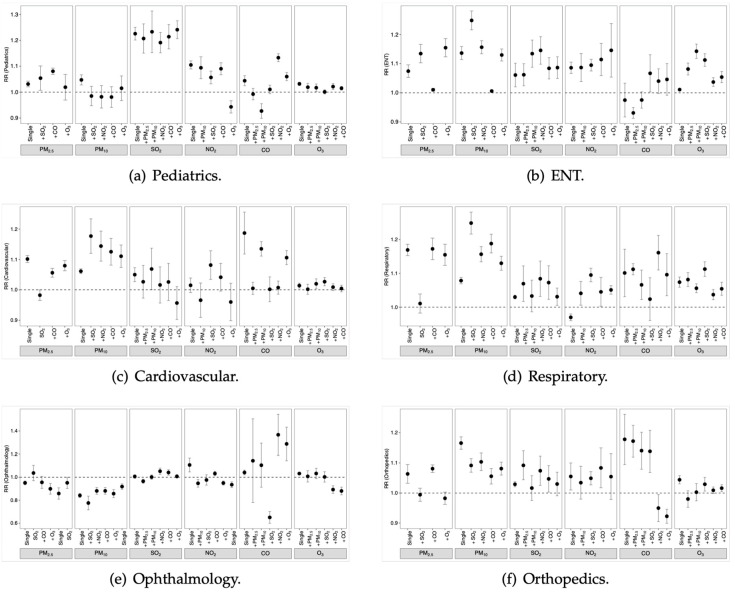
Relative risks of outpatient visits for medical departments with a 10-unit increase in air pollutant concentrations (cumulative lags from 0 to 3 days) in single- and two-pollutant models. (**a**)–(**f**): Relative risks of outpatient visits for pediatrics, ENT, cardiovascular, respiratory, ophthalmology, orthopedics, respectively.

Figure 4 shows the associations between air pollutants and outpatient visits in two-pollutant models. Because there were strong correlations between types of particulate matter, only uncorrelated pollutants ($R^{2}$ < 0.7 in Table S2) were selected for analysis. The associations between ${\mathrm{PM}}_{2.5}$ and pediatrics visits, cardiovascular visits, and respiratory visits remained significant after controlling for all other pollutants, whereas the associations between CO and pediatrics visits became insignificant after incorporating another pollutant. The association of ${\mathrm{NO}}_{2}$ and pediatrics visits decreased but remained significant after controlling for ${\mathrm{PM}}_{2.5}$ or CO and became insignificant after adding ${\mathrm{PM}}_{10}$ or ${\mathrm{SO}}_{2}$.

For outpatient visits to the cardiovascular, respiratory, and orthopedic departments, ${\mathrm{PM}}_{10}$ remained significant after controlling for other pollutants, whereas the associations between ${\mathrm{PM}}_{10}$ and visits to pediatrics and ENT became insignificant after adding ${\mathrm{SO}}_{2}$. The associations between ${\;\mathrm{SO}}_{2}$ and pediatrics, ENT, cardiovascular, and respiratory visits became insignificant, and ophthalmology became significant, after including ${\mathrm{NO}}_{2}$, whereas including ${\mathrm{PM}}_{10}$ significantly enhanced the effect of ${\mathrm{SO}}_{2}$ on ENT visits.

The associations between ${\mathrm{NO}}_{2}$ and orthopedics visits remained robust after controlling for other pollutants, whereas the associations between ${\mathrm{NO}}_{2}$ and other department visits became insignificant after including ${\mathrm{PM}}_{10}$. The effect of CO on pediatrics visits became insignificant after controlling for other pollutants, whereas the effect on orthopedics remained significant. $O_{3}$ had no significant associations with any of the departments’ visits after controlling for ${\mathrm{PM}}_{2.5}$ or ${\mathrm{SO}}_{2}$.

Sensitivity analysis showed that the associations between air-pollutant concentrations and outpatient visits for each department were not sensitive to alternative temperature lags (Figure S1) or to the use of different degrees of freedom in adjusting for long-term time trends (Figure S2).

## 4. Discussion

This study provides evidence of the effect of air pollutants on daily outpatient visits to different hospital departments in a city with moderate air pollution. The effects of air pollution on outpatient visits were different after stratification by age and season. Our research results found a significant seasonal difference in the associations between air pollutants and pediatrics department outpatient visits. Pediatrics outpatient visits were significantly sensitive to all pollutants, except that $O_{3}$, ${\mathrm{PM}}_{2.5}$, and ${\mathrm{PM}}_{10}$ had higher significant associations with pediatrics outpatient visits during the heating season than during the nonheating season. ${\mathrm{NO}}_{2}$ and CO were significantly associated with pediatric outpatients only during the heating season. These seasonal differences may be because the concentration of each pollutant was higher in the heating season due to the combustion of fossil fuels, and ${\mathrm{PM}}_{2.5}$ and ${\mathrm{PM}}_{10}$ are highly harmful after inhalation.

Our study is one of the few to have empirically analyzed the relationships between air pollution and ENT outpatient visits. In our dataset, pharyngitis and rhinitis accounted for the main ENT visits (50%), followed by thyroid diseases (38%). Compared with the findings of Zhao’s study [29] that people aged 15–65 were more likely to be affected than people over 65 years old, our results showed that ${\mathrm{PM}}_{2.5}$, ${\mathrm{PM}}_{10}$, ${\mathrm{SO}}_{2}$, and $O_{3}$ were significantly associated with ENT visits, with ${\mathrm{SO}}_{2}$ and ${\mathrm{NO}}_{2}$ being more relevant for ENT outpatients among people under 60. The results suggest that the retired population should reduce outdoor activities during working days’ commuting time and the heating season to reduce health risks and alleviate the burden on hospital systems.

Our findings indicated that air pollutants have similar associations with respiratory visits and ENT visits. These two departments have similar patient groups, and previous studies have reported associations between air pollution and respiratory outpatient visits. It is generally recognized that short-term exposure to air pollutants may increase respiratory diseases [30]. Our findings illustrated that ${\mathrm{PM}}_{2.5}$, ${\mathrm{NO}}_{2}$, ${\mathrm{SO}}_{2}$, CO, and $O_{3}$ were all associated with the respiratory outpatient visits, which were consistent with Mo et al.’s work [30]. Our data showed that COPD, lung infections, and other pulmonary diseases were major outpatient diseases, and the results showed that ${\mathrm{PM}}_{2.5}$ and ${\mathrm{NO}}_{2}$ had higher associations with respiratory outpatient visits because they adversely affected COPD and lung infections. The findings also showed that ${\mathrm{PM}}_{10}$ was not significantly associated with respiratory outpatient visits, which may be because the smaller the diameter of the particulate matter, the deeper it enters the respiratory tract. Compared with ${\mathrm{PM}}_{10}$, ${\mathrm{PM}}_{2.5}$ is more likely to accumulate in the lower respiratory tract [31]. Our results were also consistent with new research findings on COVID-19. Zhu et al. found that ${\mathrm{PM}}_{2.5}$, ${\mathrm{PM}}_{10}$, CO, ${\mathrm{NO}}_{2}$, and $O_{3}$ were significantly and positively correlated with COVID-19, whereas an increase in the concentration of ${\mathrm{SO}}_{2}$ reduced the diagnosis rate of COVID-19 (7.79% decrease) [32]. Our results indicated that an increase in ${\mathrm{SO}}_{2}$ reduced respiratory outpatient visits (3% decrease), which is in line with Zhu’s research, showing the importance of air pollution research to health issues related to COVID-19.

Previous studies on the associations between cardiovascular outpatient visits and air pollution have been inconsistent. Our results showed that ${\mathrm{PM}}_{2.5}$ has a significant effect on cardiovascular department visits, with an RR of 1.101 (95% CI: 1.090, 1.113), but ${\mathrm{PM}}_{10}\;$ and CO were also associated with cardiovascular department visits. Previous studies found evidence of a stronger association with particulate matter in the cold season (October–March) [17,33]. Samoli et al. proposed that PM in the warm season (April–September) was more likely to affect cardiovascular visits [25,34]. Our study found that ${\mathrm{PM}}_{2.5}$ had a higher significant association with cardiovascular visits during the nonheating season (March–November) than during the heating season (December–February). The difference was significant, consistent with the findings of Liu et al., who found that during a nonheating period, the effect of air pollution on CVD mortality was 2.8 times greater, and the effect was more significant for gaseous pollutants than for particulate matter in a heavily polluted city [35]. During the nonheating season, ${\mathrm{SO}}_{2}$, CO, and $O_{3}$ had significant effects on cardiovascular visits, and the effect was greater for ${\mathrm{SO}}_{2}$ than for ${\mathrm{PM}}_{2.5}$. The different effects of ${\mathrm{SO}}_{2}$ and ${\mathrm{PM}}_{2.5}$ may be due to gaseous pollutants’ physical form, which may be more likely to be inhaled into the respiratory tract and enter blood circulation, leading to dyspnea and hypoxia symptoms in CVD patients.

Our results also suggested that air pollution affected eye diseases. The present study found that ${\mathrm{PM}}_{10}$ and CO had the most significant correlations with the number of ophthalmologic visits. In contrast, the increase in $O_{3}$ concentration decreased the risk of ophthalmologic visits, especially during the nonheating season. ${\mathrm{SO}}_{2}$ had a significant effect on people under 60 years old, whereas ${\mathrm{NO}}_{2}$ and $O_{3}$ showed significant differences between age groups. Our study data showed that the ophthalmologic visits were primarily for treatment of conjunctivitis, glaucoma, dry eye, and cataract. The present results are in agreement with those of Bourcier’s study, which discovered that high levels of air pollution in Paris were linked to short-term increases in the number of people visiting ophthalmological emergency departments [36]. Our results are consistent with those of a study in Hangzhou showing that increased concentrations of CO, ${\mathrm{NO}}_{2}$, and ${\mathrm{SO}}_{2}$ were associated with an increase in the number of visits for conjunctivitis patients [37].

In this paper, the orthopedics department was used as the control for other departments. Currently, few studies have focused on orthopedic diseases, and our study found that although most of the pollutants were not relevant to orthopedic outpatient visits, ${\mathrm{PM}}_{2.5}$ and ${\mathrm{NO}}_{2}$ were significantly associated with increased orthopedic outpatient visits for people under 60 years old. The findings were exciting correlations between air pollution and orthopedic visits, but they may be due to chance, and we cannot explain them reasonably in this study.

In this paper, we found that the pollutant concentration triggers followed the exposure–response curves of various departmental outpatients and air pollutants (Figure S3). The exposure concentration triggers of ${\mathrm{PM}}_{2.5}$ in pediatric patients (61 $\mu g/m^{3}$) and cardiovascular patients (65 $\mu g/m^{3}$) were lower than the current daily air-quality standards (150 $\mu g/m^{3})$ in China (CNNAQ II), the ${\mathrm{NO}}_{2}$ exposure concentration trigger was lower than the standard (80 $\mu g/m^{3}$) for cardiovascular (70 $\mu g/m^{3}$) and respiratory (40 $\mu g/m^{3}$) patients, and the CO exposure concentration trigger was lower than the standard (4 $\mathrm{mg}/m^{3}$) for pediatric patients (2.8 $\mathrm{mg}/m^{3}$). This finding suggests that people also need to pay attention to the standard air pollution level.

The present study had several limitations. First, we used outpatient visit data from only one hospital. Although the hospital accounted for a large portion of the medical visits in Xi’an, the results are not necessarily representative. Second, we took an average of the air-pollutant concentrations from 13 fixed monitoring sites, which might have led to underestimating the associations because pollution concentrations vary geographically. Third, wind direction, wind speed, and other meteorological factors were not included in our model due to data curation issues; in future studies, they should be considered. Fourth, influenza data were not included in the model. One reason was ethical. We did not have access to detailed EMR data and could not know the exact number of admissions due to influenza. Another reason was that CDC data were for the entire Xi’an city and not for a specific hospital or region, so the influenza data could not be matched. Future studies will focus on the specific disease, such as influenza, asthma, and so on.

Overall, our findings proved that the effects of air pollutants on various departments’ outpatient visits were different. One possible advantage of this study was the inclusion of outpatient visits from various medical departments instead of the approach used in previous studies, which focused on only one specific disease. Our approach provided a more comprehensive general analysis of the effects of air pollutants. This study also provided evidence that may help hospital managers make better workload allocation decisions for different outpatient departments under different air pollution conditions.

## Figures and Tables

**Table 1:** Descriptive results for data on outpatient visits, air pollutants, and meteorological conditions.

Variable	Group	Mean ± SD	Min	Median	Max	IQR
**Daily Outpatient Visits**						
Pediatrics		322 ± 13.89	130	312	654	106.5
ENT		241 ± 50.08	102	239	396	74
Cardiovascular		269 ± 60.53	93	263	482	115
Respiratory		246 ± 60.53	108	245	434	90.5
Ophthalmology		195 ± 47	10	197	338	58.5
Orthopedics		239 ± 62.19	100	242	443	75
ENT	Heating ^1^	236 ± 53	102	232	379	79
	Nonheating ^1^	241 ± 48.43	110	241	396	66
Cardiovascular	Heating	275 ± 74.3	93	268	482	126
	Nonheating	266 ± 73.76	102	260	464	109
Respiratory	Heating	257 ± 60.63	108	257	434	87
	Nonheating	238 ± 59.2	113	237	379	90
Ophthalmology	Heating	157 ± 73.59	20	176	288	81
	Nonheating	163 ± 83.26	10	186	338	105
Orthopedics	Heating	238 ± 62.2	100	240	443	73
	Nonheating	241 ± 64.42	100	240	443	74
Pediatrics	<6	224 ± 60	102	218	460	78
	≥6	86 ± 34.99	22	78	225	47
ENT	<50	157 ± 44	10	160	259	42
	≥50	73 ± 28.54	1	74	153	42
Cardiovascular	<60	144 ± 64.76	6	153	294	106
	≥60	75 ± 51.95	13	61	219	89
Respiratory	<60	169 ± 67.46	8	188	278	71
	≥60	54 ± 32.54	10	46	130	52
Ophthalmology	<60	126 ± 58.73	2	141	264	60
	≥60	38 ± 26	1	34	142	39
Orthopedics	<50	126 ± 62.3	10	143	265	74
	≥50	82 ± 43.57	6	91	206	65
**Air pollutants**						
${\;\mathrm{PM}}_{2.5}$ ($\mu g/m^{3}$)		68.22 ± 60.17	9	47	493	51
${\mathrm{PM}}_{10}$ ($\mu g/m^{3}$)		125.9 ± 38.92	16	100	589	88
${\mathrm{SO}}_{2}$ ($\mu g/m^{3}$)		17.58 ± 11.35	4	13	71	13
${\mathrm{NO}}_{2}$ ($\mu g/m^{3}$)		54.97 ± 20.91	14	52	127	29
CO ($\mathrm{mg}/m^{3}$)		1.48 ± 0.66	0.6	1.3	5.4	0.7
$O_{3}$ ($\mu g/m^{3},$ 8 h average)		93.13 ± 57.26	6	80	280	89
**Meteorological conditions**						
Temperature (℃)		15.66 ± 9.92	−6	16.5	34	18
RH ^2^ (%)		66.12 ± 17.03	18.25	64.67	100	25.46

^1^ Heating season: from November to March; Nonheating season: from April to October. ^2^ RH, relative humidity; SD, standard deviation; Min, minimum; Max, maximum; IQR, interquartile range.

**Table 2:** Relative risks of outpatient visits with a 10-unit increase in daily air pollutant concentrations (cumulative lags from 0 to 3 days).

	$PM_{2.5}$	$PM_{10}$	$SO_{2}$	$NO_{2}$	CO	$O_{3}$
**All**						
Entire period	1.088 (0.881, 1.277)	1.035 (0.945, 1.126)	1.381 (0.795, 1.966)	1.137 (0.201, 2.047)	1.114 (0.876, 1.352)	1.152 (0.958, 1.345)
Heating	1.047 (0.890, 1.204)	1.077 (0.918, 1.236)	1.219 (1.018, 1.421)	0.930 (0.740, 1.119)	1.156 (0.634, 1.678)	1.018 (0.816, 0.816)
Nonheating	0.996 (0.828, 1.164)	0.992 (0.980, 1.004)	1.250 (1.182, 1.318)	0.784 (0.699, 0.868)	1.202 (1.025, 1.379)	0.954 (0.943, 0.965)
*p*-value	0.002	0.401	0.583	0.188	0.014	0.046
	$PM_{2.5}$		$SO_{2}$	$NO_{2}$	CO	$O_{3}$
**Pediatrics**						
Entire period	1.032 (0.998, 1.064)	1.047 (1.028, 1.067)	1.236 (1.201, 1.250)	1.105 (1.090, 1.121)	1.044 (1.025, 1.064)	1.032 (1.026, 1.039)
Heating	1.067 (1.035, 1.098)	1.009 (1.006, 1.013)	1.266 (1.236, 1.296)	1.006 (0.974, 1.038)	1.139 (1.053, 1.224)	1.098 (0.943, 1.253)
Nonheating	1.008 (0.975, 1.040)	0.959 (0.943, 0.975)	0.995 (0.936, 1.055)	1.130 (1.047, 1.214)	0.843 (0.801, 0.886)	0.979 (0.970, 0.988)
*p*-value	0.134	0.001	<0.001	<0.001	<0.001	0.856
Age < 6	1.078 (0.972, 1.185)	1.038 (1.014, 1.062)	1.218 (1.049, 1.388)	1.024 (0.76, 1.289)	0.983 (0.833, 1.132)	1.077 (0.902, 1.251)
Age ≥ 6	1.01 (1.002, 1.018)	1.005 (0.978, 1.033)	1.196 (0.903, 1.489)	1.057 (1.006, 1.108)	1.083 (0.867, 1.299)	0.995 (0.861, 1.129)
*p*-value	0.425	0.944	0.0002	<0.01	0.0005	0.134
	$PM_{2.5}$	$PM_{10}$	$SO_{2}$	$NO_{2}$	CO	$O_{3}$
**ENT**						
Entire period	1.074 (1.053, 1.096)	1.136 (1.114, 1.159)	1.061 (1.021, 1.102)	1.086 (1.067, 1.106)	0.975 (0.9170, 1.032)	1.011 (1.007, 1.015)
Heating	1.010 (0.993, 1.082)	1.063 (1.034, 1.091)	0.916 (0.890, 0.941)	1.129 (1.094, 1.164)	1.051 (1.014, 1.088)	1.185 (1.144, 1.226)
Nonheating	0.841 (0.709, 0.973)	1.029 (1.001, 1.057)	1.118 (0.949, 1.287)	1.104 (0.898, 1.310)	1.050 (0.815, 1.285)	1.020 (0.999, 1.042)
*p*-value	<0.001	0.066	0.103	0.337	0.570	<0.001
Age < 50	1.085 (1.067, 1.102)	1.006 (0.998, 1.014)	1.105 (1.062, 1.148)	1.02 (0.794, 1.245)	0.916 (0.574, 1.259)	1.036 (0.871, 1.201)
Age ≥ 50	1.003 (0.967, 1.04)	1.162 (1.065, 1.259)	0.927 (0.687, 1.166)	1.011 (0.808, 1.213)	0.989 (0.668, 1.309)	0.963 (0.836, 1.090)
*p*-value	0.274	0.003	0.007	0.031	0.121	0.133
	$PM_{2.5}$	$PM_{10}$	$SO_{2}$	$NO_{2}$	CO	$O_{3}$
**Cardiovascular**						
Entire period	1.101 (1.090, 1.113)	1.061 (1.053, 1.069)	1.050 (1.027, 1.073)	1.015 (0.974, 1.022)	1.187 (1.139, 1.243)	1.014 (1.006, 1.022)
Heating	0.991 (0.977, 1.006)	0.967 (0.946, 0.988)	1.339 (1.289, 1.389)	0.946 (0.905, 0.987)	1.012 (0.999, 1.026)	1.273 (1.189, 1.358)
Nonheating	1.138 (0.888, 1.388)	1.014 (1.006, 1.023)	1.306 (1.097, 1.515)	0.931 (0.879, 0.984)	1.053 (0.487, 1.62)	1.026 (1.020, 1.032)
*p*-value	0.001	<0.001	0.095	0.565	0.008	<0.001
Age < 60	1.134 (1.023, 1.245)	1.046 (0.991, 1.101)	1.027 (0.935, 1.119)	1.068 (1.029, 1.107)	1.111 (0.843, 1.379)	1.051 (0.964, 1.137)
Age ≥ 60	1.126 (0.996, 1.256)	1.149 (1.071, 1.229)	1.184 (1.062, 1.307)	1.023 (0.901, 1.144)	0.971 (0.912, 1.029)	1.017 (0.964, 1.071)
*p*-value	0.062	0.342	<0.001	0.07	<0.001	0.003
	$PM_{2.5}$	$PM_{10}$	$SO_{2}$	$NO_{2}$	CO	$O_{3}$
**Respiratory**						
Entire period	1.169 (1.152, 1.186)	1.078 (1.069, 1.088)	0.970 (0.961, 0.981)	1.030 (1.024, 1.035)	1.101 (1.031, 1.171)	1.074 (1.059, 1.089)
Heating	1.022 (1.007, 1.037)	1.037 (1.018, 1.056)	0.993 (0.966, 1.020)	1.026 (1.011, 1.042)	1.151 (1.044, 1.258)	1.112 (0.942, 1.281)
Nonheating	1.143 (1.085, 1.201)	1.030 1.007, 1.054)	0.992 (0.954, 1.041)	1.081 (1.029, 1.133)	1.078 (1.040, 1.116)	1.015 (1.005, 1.024)
*p*-value	<0.001	0.488	0.087	<0.001	<0.001	0.604
Age < 60	1.019 (0.789, 1.249)	1.028 (0.862, 1.195)	1.012 (1.001, 1.023)	1.170 (1.161, 1.182)	1.077 (1.028, 1.126)	1.101 (0.962, 1.242)
Age ≥ 60	0.994 (0.949, 1.039)	1.060 (0.995, 1.083)	0.914 (0.724, 1.104)	1.098 (1.087, 1.109)	1.086 (0.979, 1.193)	1.011 (1.003, 1.019)
*p*-value	0.181	0.039	0.155	<0.01	0.101	0.045
	$PM_{2.5}$	$PM_{10}$	$SO_{2}$	$NO_{2}$	CO	$O_{3}$
**Ophthalmology**						
Entire period	1.023 (1.003, 1.044)	1.098 (1.079, 1.117)	1.006 (1.009, 1.013)	1.034 (1.023, 1.046)	1.078 (1.033, 1.123)	0.973 (0.927, 1.018)
Heating	1.014 (0.989, 1.041)	1.074 (1.053, 1.096)	1.068 (1.028, 1.107)	1.148 (1.001, 1.296)	1.064 (1.044, 1.085)	1.227 (1.168, 1.285)
Nonheating	1.080 (1.030, 1.130)	1.024 (1.020, 1.028)	0.918 (0.806, 1.029)	1.042 (1.034, 1.050)	0.906 (0.886, 0.925)	0.930 (0.905, 0.956)
*p*-value	0.019	0.453	0.275	0.045	0.038	<0.001
Age < 60	1.037 (0.995, 1.079)	0.995 (0.949, 1.041)	1.086 (1.023, 1.148)	0.914 (0.724, 1.104)	1.144 (1.104, 1.183)	1.016 (1.008, 1.024)
Age ≥ 60	1.027 (1.018, 1.037)	1.020 (0.790, 1.251)	1.077 (0.933, 1.221)	1.056 (1.015, 1.097)	1.154 (1.093, 1.214)	1.100 (0.961, 1.239)
*p*-value	0.451	0.228	<0.001	0.005	0.215	<0.001
	$PM_{2.5}$	$PM_{10}$	$SO_{2}$	$NO_{2}$	CO	$O_{3}$
**Orthopedics**						
Entire period	1.063 (1.032, 1.095)	1.166 (0.989, 1.342)	1.029 (0.976, 1.082)	1.178 (1.092, 1.263)	1.055 (0.969, 1.140)	1.044 (0.958, 1.129)
Heating	0.985 (0.976, 0.993)	1.035 (1.006, 1.064)	0.935 (0.902, 0.967)	1.123 (1.029, 1.218)	1.064 (1.044, 1.085)	1.106 (1.009, 1.203)
Nonheating	0.987 (0.828, 1.146)	1.008 (0.995, 1.020)	1.057 (1.030, 1.083)	1.207 (1.076, 1.338)	1.176 (0.818, 1.533)	1.034 (1.020, 1.049)
*p*-value	0.938	0.025	<0.001	0.275	0.238	0.938
Age < 60	1.056 (1.036, 1.076)	1.122 (1.107, 1.137)	0.996 (0.841, 1.150)	1.039 (0.973, 1.104)	0.990 (0.761, 1.219)	0.974 (0.875, 1.073)
Age ≥ 60	1.038 (1.005, 1.070)	1.096 (1.085, 1.107)	1.012 (0.843, 1.180)	1.112 (1.013, 1.212)	1.142 (0.881, 1.405)	1.001 (0.898, 1.104)
*p*-value	0.286	0.779	0.051	0.009	0.689	0.764

## Data Availability

The air pollution data that support the findings of this study are available on request from the corresponding author, Dr. Bo Kou. The AAD outpatient visits data are not publicly available due to the privacy restrictions.

## Supplementary Materials

The following supporting information can be downloaded at: https://www.mdpi.com/article/10.3390/toxics11020166/s1. Figure S1. Associations of air pollutants with department visits using different lags of temperature. Figure S2. Associations of air pollutants with department visits using different degrees of freedom for time trends. Figure S3. Concentration-response relationship curves for the associations between short-term exposure to air pollutants (cumulative lags from 0 to 3 days) and outpatient visits of different departments. Table S1. Spearman’s correlation coefficients between daily air pollutant concentrations and meteorological conditions in Xi’an, 2016–2018. Table S2. Correlation coefficients between daily air pollutant concentrations in Xi’an, 2016–2018.
