# Supplementary material for: Short-Term Effect of Moderate Level Air Pollution on Outpatient Visits for Multiple Clinic Departments: A Time-Series Analysis in Xi’an China
Source: Toxics. 2023 Feb 9;11(2):166. doi: 10.3390/toxics11020166 (PMC9967132; doi:10.3390/toxics11020166)
Supplement: Supplementary file 1 [file toxics-11-00166-s001.zip › toxics-2136796-supplementary.pdf]

Supplementary Material

Short-Term Effect of Moderate Level Air Pollution on Outpatient Visits for Multiple Clinic Departments: A Time-Series Analysis in Xi'an China

Qingnan Wang [1], Zhuo Chen [2,3], Wei Huang [1,4], Bo Kou [5,*] and Jingwei Li [1]

1 Department of Information Management, School of Management, Xi'an Jiaotong University, Xi'an 710049, China

2 College of Public Health, University of Georgia, Athens, GA 30602, USA

3 School of Economics, University of Nottingham Ningbo China, Ningbo 315000, China

4 College of Business, Southern University of Science and Technology, Shenzhen 518055, China

5 Department of Otolaryngology, the First Affiliated Hospital of Xi'an Jiaotong University, Xi'an 710016, China

* Correspondence: bobiekou84@gmail.com

Caption list

Figure S1. Associations of air pollutants with department visits using different lags of temperature.

Figure S2. Associations of air pollutants with department visits using different degrees of freedom for time trends.

Figure S3. Concentration-response relationship curves for the associations between short-term exposure to air pollutants (cumulative lags from 0 to 3 days) and outpatient visits of different departments.

Table S1. Spearman's correlation coefficients between daily air pollutant concentrations and meteorological conditions in Xi'an, 2016-2018.

Table S2. Correlation coefficients between daily air pollutant concentrations in Xi'an, 2016-2018.

Figures

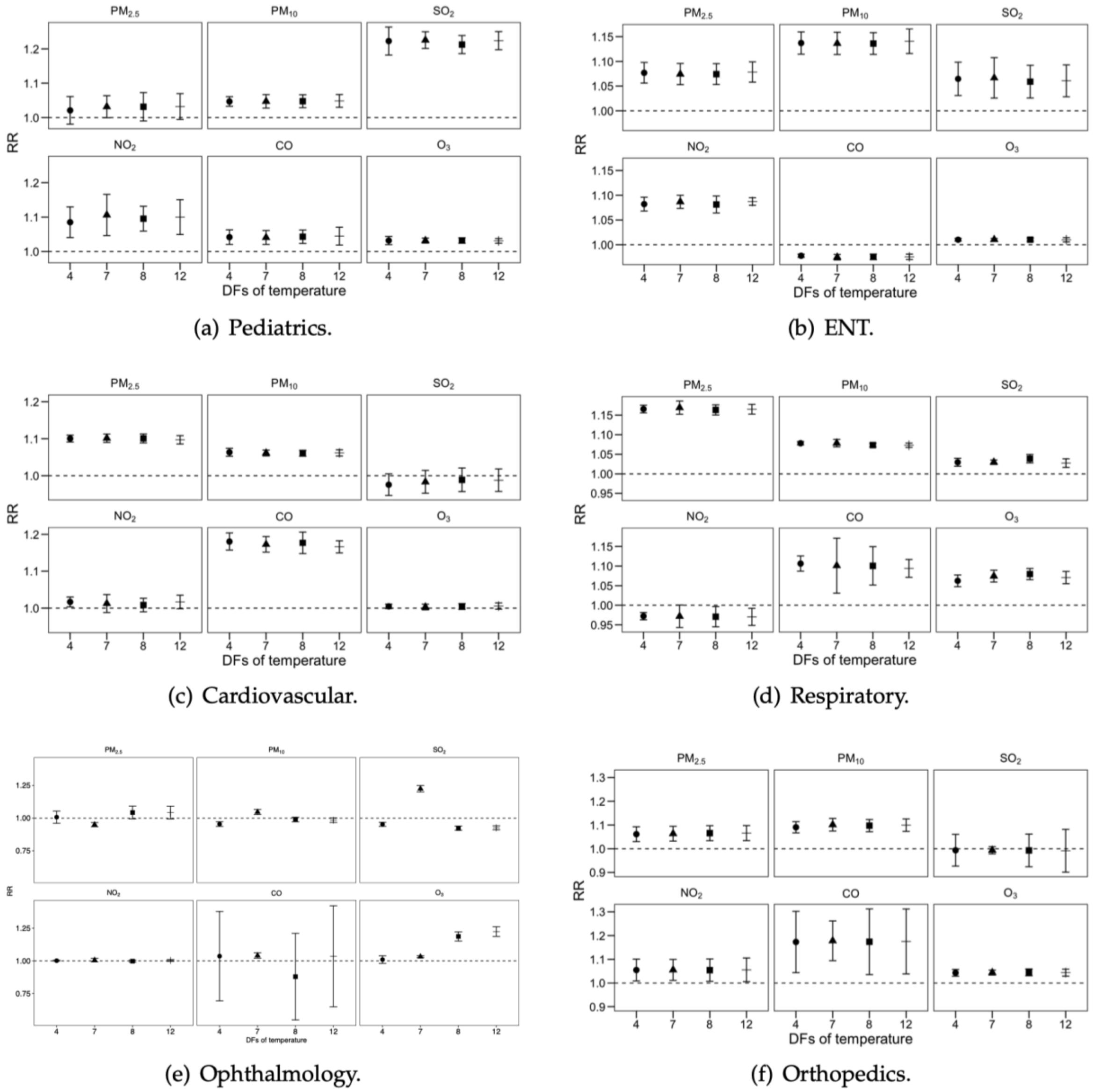

Figure S1. Associations of air pollutants with department visits using different lags of temperature.

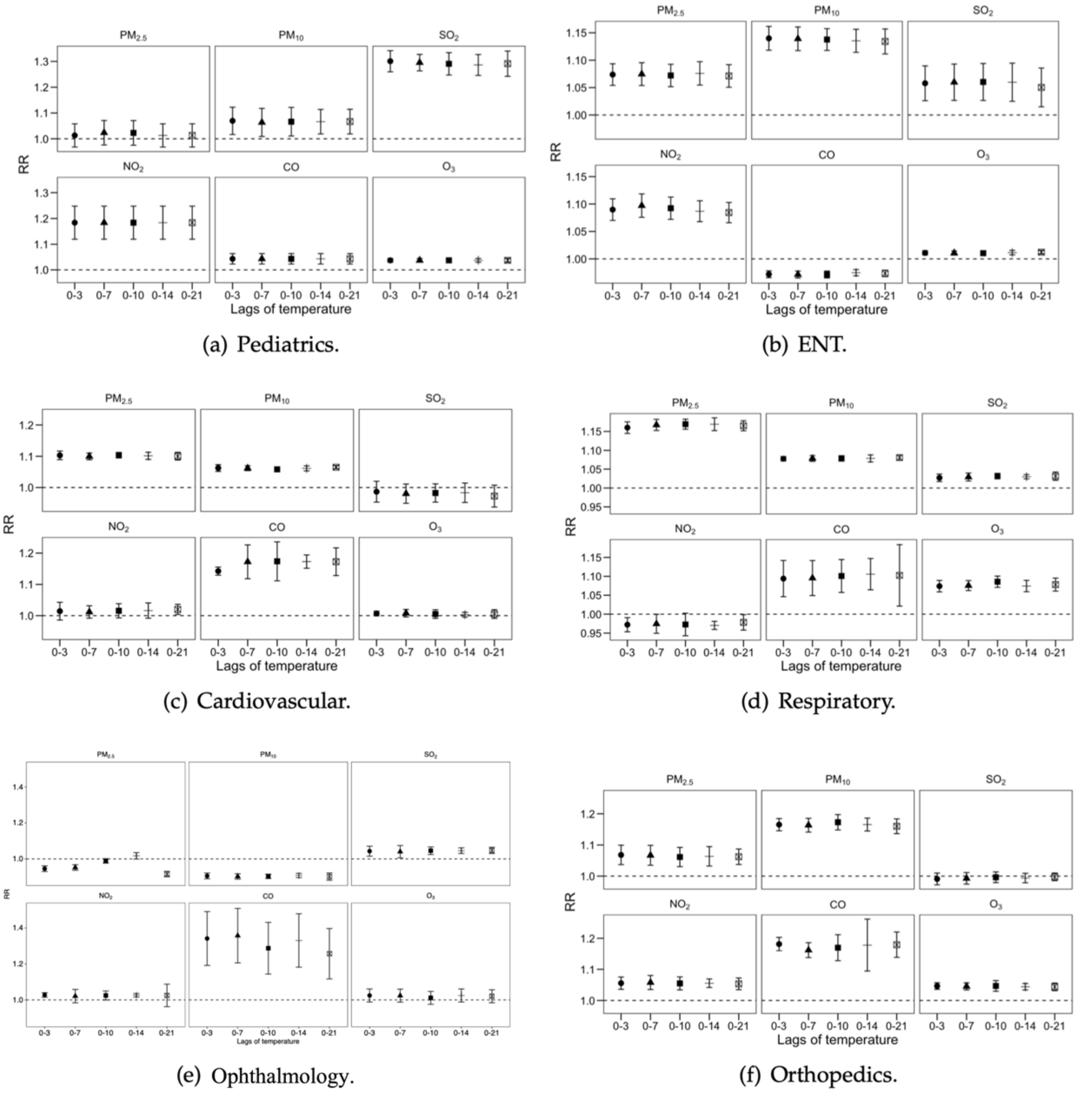

(a) Pediatrics.

(b) ENT.

(c) Cardiovascular.

(d) Respiratory.

(e) Ophthalmology.

(f) Orthopedics.

Figure S2. Associations of air pollutants with department visits using different degrees of freedom for time trends.

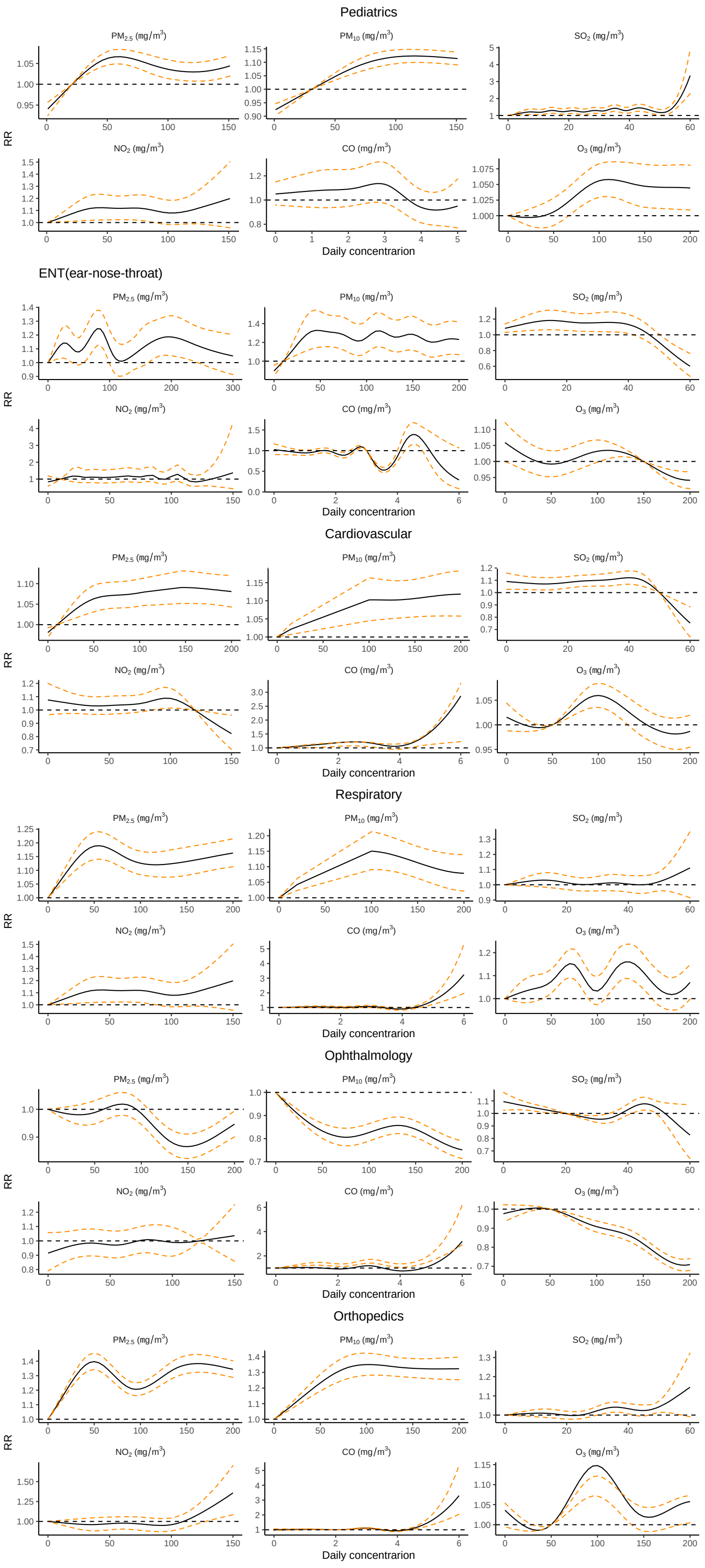

Figure S3. Concentration-response relationship curves for the associations between short-term exposure to air pollutants (cumulative lags from 0 to 3 days) and outpatient visits of different departments.

Tables

Table S1. Spearman's correlation coefficients between daily air pollutant concentrations and meteorological conditions in Xi'an, Jan.2016-Dec.2018.

	PM_{10}	SO_2	NO_2	CO	O_3	Temperature	RH
$PM_{2.5}$	0.93**	0.78**	0.86**	0.74**	-0.50**	-0.59**	0.01
PM_{10}		0.80**	0.77**	0.76&&	-0.41**	0.52**	-0.16**
SO_2			0.77**	0.82**	-0.49**	-0.70**	-0.28**
NO_2				0.71**	-0.39**	-0.51**	-0.18**
CO					-0.58**	-0.64**	0.08**
O_3						0.81**	-0.36**
Temperature							-0.02

RH: relative humidity.
**: p value<0.01.

Table S2. Correlation coefficients between daily air pollutant concentrations in Xi'an, Jan.2016-Dec.2018.

R^2	PM_{10}	SO_2	NO_2	CO	O_3
$PM_{2.5}$	0.85	0.61	0.74	0.55	0.26
PM_{10}		0.66	0.61	0.61	0.17
SO_2			0.59	0.67	0.23
NO_2				0.50	0.32
CO					0.14
